# Electroencephalography in delirium assessment: a scoping review

**DOI:** 10.1186/s12883-022-02557-w

**Published:** 2022-03-11

**Authors:** Tim L. T. Wiegand, Jan Rémi, Konstantinos Dimitriadis

**Affiliations:** 1 cBRAIN, Department of Child and Adolescent Psychiatry, Psychosomatic and Psychotherapy, Ludwig-Maximilians-Universität, Munich, Germany; 2 Department of Neurology, University Hospital, Ludwig Maximilian University, 15 Marchioninistr, 81377 Munich, Germany; 3 Institute for Stroke and Dementia Research (ISD), University Hospital, Ludwig Maximilians University, Munich, Germany

**Keywords:** Intensive care, Delirium, EEG, Electrophysiology, Dementia, Epilepsy

## Abstract

**Background:**

Delirium is a common disorder affecting around 31% of patients in the intensive care unit (ICU). Delirium assessment scores such as the Confusion Assessment Method (CAM) are time-consuming, they cannot differentiate between different types of delirium and their etiologies, and they may have low sensitivities in the clinical setting. While today, electroencephalography (EEG) is increasingly being applied to delirious patients in the ICU, a lack of clear cut EEG signs, leads to inconsistent assessments.

**Methods:**

We therefore conducted a scoping review on EEG findings in delirium. One thousand two hundred thirty-six articles identified through database search on PubMed and Embase were reviewed. Finally, 33 original articles were included in the synthesis.

**Results:**

EEG seems to offer manifold possibilities in diagnosing delirium. All 33 studies showed a certain degree of qualitative or quantitative EEG alterations in delirium. Thus, normal routine (rEEG) and continuous EEG (cEEG) make presence of delirium very unlikely. All 33 studies used different research protocols to at least some extent. These include differences in time points, duration, conditions, and recording methods of EEG, as well as different patient populations, and diagnostic methods for delirium. Thus, a quantitative synthesis and common recommendations are so far elusive.

**Conclusion:**

Future studies should compare the different methods of EEG recording and evaluation to identify robust parameters for everyday use. Evidence for quantitative bi-electrode delirium detection based on increased relative delta power and decreased beta power is growing and should be further pursued. Additionally, EEG studies on the evolution of a delirium including patient outcomes are needed.

**Supplementary Information:**

The online version contains supplementary material available at 10.1186/s12883-022-02557-w.

## Introduction

The Diagnostic and Statistical Manual of Mental Disorders, 5th Edition (DSM-5), defines delirium as a clinical syndrome with acute disturbances in consciousness, attention, and awareness [1]. Typical etiologies are substance intoxication or withdrawal, post-surgery effects, or other causes of acute brain dysfunction or encephalopathy [1]. With regard to its psychomotor manifestation, hyperactive, hypoactive, or mixed types of delirium can be distinguished [1]. Delirium is a common disorder, especially in the emergency department, with a strong association with patient age and disease severity [2, 3]. A meta-analysis from 2018 reported a pooled prevalence of delirium of 31% of patients in the intensive care unit (ICU), and a pooled incidence of 22% of ICU patients [4]. However, the incidence of delirium in the ICU has been reported to be as high as 82% of ICU patients [5]. Among ICU and non-ICU patients, delirium is associated with higher risk of complications and mortality [6–10], long-term cognitive impairment [10–13], extended length of hospital stays [14], and increased rate of institutionalization after discharge [6, 15, 16].

Despite the frequency and impact of delirium, it often remains underdiagnosed or insufficiently documented by physicians in the ICU or the recovery room [2, 17–20]. Additionally, the diagnostic process, using the DSM-5 or the International Statistical Classification of Diseases and Related Health Conditions, 10th Edition (ICD-10) criteria depends on the clinical experience of the rating physician [17, 19, 21, 22]. Thus, several delirium assessment-tools have been developed. Among these are the Confusion Assessment Method (CAM) [23] and its adaption for the ICU (CAM-ICU) [5], the Intensive Care Delirium Screening Checklist (ICDSC) [24], the Nursing Delirium Screening Scale (Nu-DESC) [25], and the Delirium Rating Scale (DRS) [26, 27]. The reported sensitivities and specificities vary greatly for these tools. For example, for the CAM/CAM-ICU, three meta-analyses reported sensitivities around 78% and specificities around 97% [28–30]. However, in the clinical setting, one study reported a sensitivity of 47%, while the specificity was 98% [31].

Especially in presence of neurological symptoms overlapping with those of delirium, a screening tool with both a high sensitivity and a high specificity is needed [32]. In addition, even with delirium assessment tools, the diagnosis of delirium is time-consuming. However, precise, objective, and consistent biomarkers are yet unavailable, which may explain the current lack of standardized approaches [33, 34].

Electroencephalography (EEG) may be a promising tool for providing diagnostic biomarkers that could improve diagnostic accuracy in delirium [35]. Previous systematic review articles have pointed towards the utility of EEG in differentiating delirious and non-delirious individuals [36, 37]. More specifically, Boord et al. [36] found that EEG slowing and reduced functional connectivity allow to differentiate both groups. Van der Kooi et al. [37] report that relative alpha and theta power most often allowed distinguishing delirious and non-delirious patients. However, while EEG is increasingly applied to delirious patients in the ICU, a lack of clear clinical and research guidelines as well as definite EEG signs (like epileptiform discharges for epilepsy) leads to inconsistent evaluations. This article therefore aims to review and assess EEG findings in delirium presented in the literature as well as clinical or research protocols. More specifically, we aim to review the sensitivity and specificity of routine (rEEG) and continuous EEG (cEEG) for detecting delirium, choice of electrodes, possible influence of confounding factors, as well as the role of epilepsy and sleep patterns in delirium.

## Methods

### Literature search and study selection

We followed the Preferred Reporting Items for Systematic Reviews and Meta-Analyses extension for Scoping Reviews (PRISMA-ScR) guidelines [38]. Database search was conducted on Embase and PubMed in April 2021 by two reviewers (T.L.T.W. and K.D.). The strategy combined MeSH-terms, where applicable, with non-MeSH. For the exact search terms, please see supplementary materials. Inclusion criteria for the literature were A) evaluating EEG in diagnosis of delirium; B) use of EEG in treatment optimization; C) a population of 18 years or older; and D) publications written in English. In addition, exclusion criteria were A) studies in a language other than English; B) review articles, commentaries, editorials, case studies, and studies with no original data; and C) articles with EEG signals that were modified or already interpreted (e.g., using bispectral index), and that did not report EEG signals.

**Fig. 1:**
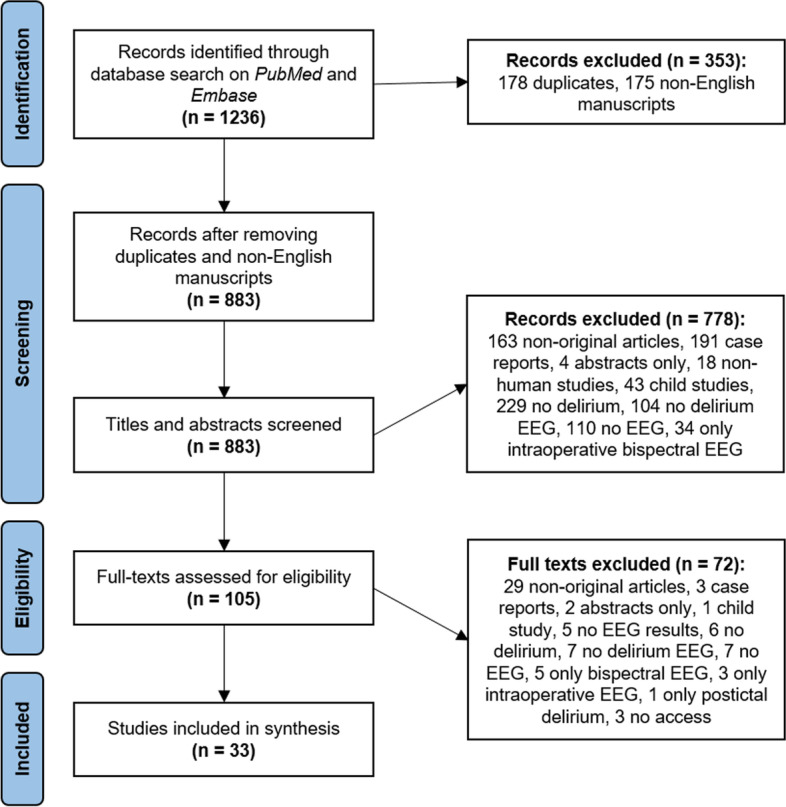
Literature search process (EEG = electroencephalography)

The database search provided a total of 1236 articles (please see Fig. 1). After removing papers in a language other than English as well as duplicates, 883 articles remained. These were screened for titles and abstracts, and a total of 778 articles were excluded. The remaining 105 articles were screened with regard to their full text, and a total of 72 articles were excluded. The remaining 33 articles were the final tally and were further analyzed. The reasons for exclusion for each article were documented. Consensus on discrepancies was reached through discussion. Finally, all authors agreed on which articles should be included.

### Quality assessment

The methodological quality of studies was assessed using a QUADAS-2-based rating [39]. The QUADAS-2 assesses the risk of bias in a study, and its applicability to the research question. Risk of bias was evaluated based on the four domains A) patient selection; B) index test (i.e., EEG); C) reference standard (i.e., delirium diagnostics); and D) flow and timing of the study. For each of these domains three or four signaling questions were defined based on which a summary score was calculated. For details on the signaling questions, please see supplementary materials. Applicability to the research question was evaluated based on the three domains A) patient selection; B) index test; and C) reference standard. Inter-rater reliability was calculated with Cohen’s Kappa [40].

### Data extraction and synthesis

Two authors (T.L.T.W. and K.D.) performed the extraction of the following data: study characteristics (retrospective/prospective design, sample size), patient demographics (age, sex/gender, admission diagnosis, dementia, alcohol/substance abuse, relevant medication), delirium diagnosis and assessment, neuroimaging, laboratory values, timing and setting of EEG, EEG analysis method, and major EEG findings. Consensus on discrepancies was reached through discussion. Finally, all authors agreed on which data were relevant.

Due to the heterogeneity of methods and presentation of results, a statistical analysis or meta-analysis was not possible. Thus, for synthesis of findings, a narrative approach based on the methodology described by Popay et al. [41] was used. For clarity, we consistently use 10-20 electrode designations.

## Results

For a summary of results, please see Table 1.

**Table 1:** Summary of results

Authors	Journal	Design	N Total (Delirium/No Delirium)	Mean Age	% Female	Admission	Dementia	Substance (Ab)use	Delirium Tool	EEG Type	Ana-lysis Meth-od	EEG Set-up	Summary Findings
Allahyari et al. (1976) [42]	Psychiatr Clin	Prospective	30 (30/0)	N.m.	0	Intoxication/ Withdrawal	N.m.	Substance abuse reported and considered in analysis	N.m.	rEEG	Qualitative	10-20 system	Most patients showed a physiological EEG; 1 patient showed generalized paroxysmal spike wave bursts; 4 patients showed diffuse slow activity during delirium tremens, partly accompanied by rhythmic bilateral slow waves
Azabou et al. (2015) [43]	PLoS One	Prospective	110 (22/88)	63,8	29	Sepsis	N.m.	Substance abuse reported but NOT considered in analysis	CAM	rEEG	Qualitative	10-20 system, 13 channels	Absence of EEG reactivity, delta-predominant background, PDs, Synek grade ≥ 3, and Young grade > 1 at day 1 to 3 after admission were predictors of ICU mortality and associated with delirium; ESZ and PDs in about 20% of all patients
Evans et al. (2017) [44]	Clin Neurophysiol	Prospective	12 (3/9)	66,8	42	Surgery	No subjects with dementia	No patients with substance abuse	CAM, DRS	cEEG	Quantitative	0.1 Hz high pass filter, 70 Hz low pass filter, EMG	Diminished total sleep time and longer latency to sleep onset during first night in hospital associated with greater delirium severity on day 2 after surgery; Delirious patients slept 2.4 h less and took 2 h longer to fall asleep than non-delirious patients; Greater waking EEG delta power on day 1 after surgery and less non-REM sleep EEG delta power on night 2 predicted delirium severity on day 2 after surgery
Fleischmann et al. (2019) [45]	Clin EEG Neurosci	Retrospective	376 (31/345)	75,3	39	Mixed	Mixed collective - dementia NOT considered in analysis	N.m.	CAM	rEEG	Quantitative	10-20 system, 256 Hz sampling rate, 70 Hz low pass filter, 50 Hz notch filter	Differentiation of delirious patients vs. normal controls using spectral power at F3-P4 at 2 Hz and C3-O1 at 19 Hz achieved 100% sensitivity and 99% specificity
Fleischmann et al. (2019) [45]	Pilot Feasibility Stud	Retrospective	543 (129/414)	73,6	43	Mixed	N.m.	N.m.	DSM/ ICD	rEEG	Quantitative	10-20 system, 256 Hz sampling rate; 50 Hz discrete FT filter	Significant differences in delirious and non-delirious patients in EEG power, connectivity, and network characteristics; Global alpha and regional beta band disconnectivity as well as theta band hyperconnectivity in delirious patients; Abnormalities affected networks engaged in consciousness, attention, memory, executive control, and salience detection
Hunter et al. (2020) [46]	AIMS Neurosci	Prospective	10 (5/5)	63,8	20	Mixed	No subjects with dementia	N.m.	CAM	rEEG	Quantitative	10-20 system, 0.16-52 Hz band pass filter, 9 channels	EEG slowing as well as general loss of directed functional connectivity between recording sites in delirious patients; 3 electrodes were sufficient to differentiate groups, with significantly higher slow-to-fast frequency power ratio in delirious compared to non-delirious patients in C3, P3, T7, or all 3
Jacobson et al. (1993) [47]	J Neurol Neurosurg Psychiatry	Retrospective	34 (18/16)	76,6	65	Mixed	Mixed collective - dementia considered in analysis	No patients with substance abuse	DSM/ ICD	rEEG	Qualitative & quantitative	10-20 system, 16 channels + eye movement	Differentiation of normal vs. encephalopathic records using MMSE scores and relative power in the alpha frequency (up to 94% correctly classified; no sensitivity/specificity provided); Differentiation of patients with delirium vs. patients with dementia using EEG theta activity, relative power in delta, and brain map rating (up to 93% correctly classified)
Jacobson et al. (1993) [47]	Biol Psychiatry	Prospective	33 (15/18)	75	73	N.m.	Mixed collective - dementia considered in evaluation	No patients with substance abuse	DSM/ ICD	rEEG	Qualitative & quantitative	10-20 system, 16 channels + eye movement	In delirious patients, changes in score of relative power map and changes in relative power in the alpha band significantly associated with changes in MMSE; In patients with dementia only, changes in score for absolute power maps and changes in absolute power in the delta band were significantly associated with changes in MMSE
Katz et al. (1991) [48]	Int Psychogeriatr	Prospective	28 (10/18)	N.m.	N.m.	N.m.	N.m.	N.m.	N.m.	cEEG	Quantitative	10-20 system, 6 channels	Significantly differences in theta and beta power between delirious and non-delirious patients during hospitalization; Significant differences in subsequent change of theta, delta, and alpha power between delirious and non-delirious patients
Keijzer et al. (2020) [49]	Resuscitation	Prospective	141 (47/94)	61,7	16	Cardiac arrest	N.m.	N.m.	DSM/ ICD	cEEG	Quantitative	10-20 system, 500 Hz sampling rate, 0.5-30 Hz band pass filtered with Butterworth filter, 21 channels	Delirium associated with longer hospitalization, and more frequent discharge to rehabilitation center or nursing home; EEG predicted delirium with 91% specificity and 40% sensitivity
Kimchi et al. (2019) [50]	Neurology	Prospective	200 (121/79)	59,2	43	Mixed	No subjects with dementia	N.m.	CAM	rEEG	Qualitative	10-20 system	Generalized theta or delta slowing were associated with delirium; EEG slowing correlated with delirium severity; EEG slowing was associated with longer hospitalizations, worse functional outcomes, and increased mortality
Knauert et al. (2018) [51]	J Crit Care	Retrospective	93 (93/0)	56,2	48	Mixed	No subjects with dementia	N.m.	Other	cEEG	Qualitative	10-20 system, 17 channels + eye movement, EMG	Delirious patients without K-complexes or without sleep spindles had more severe encephalopathy and higher odds of death
Koponen et al. (1989) [52]	J Neurol Neurosurg Psychiatry	Prospective	70 (51/19)	73,8	57	Mixed	Mixed collective - dementia considered in analysis	No patients with substance abuse	DSM/ ICD	rEEG	Quantitative	10-20 system, 70 Hz high frequency limit, 16 channels	Significantly reduced alpha power, increased theta and delta activity and slowing of the peak and mean frequencies in delirious compared to non-delirious patients; Alpha power and various ratio parameters correlated with MMSE score, and delta percentage and mean frequency with the lengths of delirium and hospitalization
Matsushima et al. (1997) [53]	Biol Psychiatry	Prospective	20 (10/10)	53,1	20	acute myocardial infarction.	N.m.	No patients with substance abuse	DSM/ ICD	rEEG	Qualitative & quantitative	10-20 system, 16 channels + eye movement	Delirious patients showed slowing of background EEG activity, particularly on day 2 after admission, and many rapid group, and rapid superimposed on slow eye movements, particularly on day 3; From days 2 to 3, EEG showed improvement in consciousness, and eye tracking signs of anxiety and tension
Naeije et al. (2014) [54]	Epilepsy Behav	Prospective	64 (64/0)	82	70	N.m.	N.m.	N.m.	CAM	cEEG & rEEG	Qualitative	10-20 system, 21 channels	cEEG detected NCSE in 28% and focal IEDs in 16% of delirious patients; rEEG detected NCSE in 6% and focal IEDs in 16% of delirious patients; History of cognitive impairment and use of antibiotics and hypernatremia associated with NCSE; NCSE associated with longer hospitalization and higher mortality rate
Nielsen et al. (2019)	Neurocrit Care	Prospective	102 (66/36)	71	33	Mixed	N.m.	N.m.	CAM	cEEG	Qualitative & quantitative	10-20 system, 1 kHz sampling rate, 1-120 Hz band pass filter, 19 channels + eye movement, EMG	Absence of delirium associated with preserved high-frequency beta activity and cEEG reactivity; Delirium associated with preponderance of low-frequency cEEG activity and absence of high-frequency cEEG activity; Sporadic PDs in 15 patients, 13 of which were delirious; No patient showed evidence of NCSE
Numan et al. (2017) [55]	Clin Neurophysiol	Prospective	58 (18/40)	75,3	53	Surgery	N.m.	N.m.	DSM/ ICD	cEEG	Quantitative	10-20 system, 512 sampling rate, 0.15 Hz high pass filter, 70 Hz low pass filter, 21 channels	Significantly lower average PLI in patients with delirium or recovery from anesthesia compared to non-delirious patients; Loss of anterior-posterior information flow in alpha band in patients with delirium or recovery from anesthesia; Significantly lower functional connectivity in alpha band in patients with delirium or recovery from anesthesia compared to non-delirious patients; 77% sensitivity and 85% specificity in discrimination of delirious vs. non-delirious patients; 78% sensitivity and 68% specificity for patients with delirium vs. recovery from anesthesia
Numan et al. (2019) [56]	Br J Anaesth	Prospective	159 (55/104)	76,9	33	Surgery	Mixed collective - dementia NOT considered in analysis	Substance abuse reported but NOT considered in analysis	DSM/ ICD, CAM, DRS	rEEG	Quantitative	10-20 system, 512 Hz sampling rate, 50 Hz notch filter, 0.15 Hz IIR filter, 4 channels	Depending on the cut-off, relative delta power predicted delirium with up to 90% sensitivity and up to 90% specificity (AUROC: 0.75) based on just one minute artifact-free EEG recording
Plaschke et al. (2007) [57]	Anaesthesia	Prospective	37 (17/20)	63,7	27	Mixed	No subjects with dementia	No patients with substance abuse	CAM	rEEG	Quantitative	10-20 system, 0.5 Hz high pass filter, 16 channels	Significantly higher theta power and lower alpha power in delirious compared to non-delirious patients; No group differences in SAA
Reischies et al. (2005) [58]	Psychiatry Res	Prospective	12 (12/0)	56,7	58	treatment-resistant major depression	N.m.	N.m.	DSM/ ICD, DRS	rEEG	Quantitative	10-20 system, 250 Hz sampling rate, 0.15-50 Hz band pass filter, 50 Hz notch filter, 0.5 Hz high pass filter, 32 channels + eye movement	Compared to baseline, significant increases in delta and theta power and decrease in alpha power during delirium; Decrease of theta activity at Fz in following 24 h correlated with recovery of awareness and performance of free recall; Source analysis with LORETA indicated that the main generators of the theta excess during delirium were localized in the anterior cingulate cortex and right fronto-temporal areas
Sambin et al. (2019) [59]	Front Neurol	Prospective	50 (50/0)	84	66	Mixed	Mixed collective - dementia considered in analysis	Substance abuse reported and considered in analysis	CAM	cEEG	Qualitative	10-20 system, 21 channels	NCSE in 12% and interictal discharges in 30% of delirious patients
Sun et al. (2019) [60]	NPJ Digit Med	Prospective	174 (N.m./ N.m.)	N.m.	33	Mixed	No subjects with dementia	N.m.	CAM	N.m.	N.m.	N.m.	Deep learning model achieved detected delirium with 69% sensitivity and 83% specificity
Tanabe et al. (2020) [61]	Br J Anaesth	Prospective	70 (22/48)	70,4	39	Surgery	N.m.	N.m.	CAM, DRS	rEEG	Quantitative	0.1-50 Hz band pass filter with Hamming windowed-sinc FIR filter, 256 channels	Preoperatively, patients with postoperative delirium had significantly higher alpha power, higher alpha band connectivity, but impaired structural connectivity; Postoperatively, delirium was associated with increased SWA in parieto-occipital and frontal cortex, with accompanying breakdown in functional connectivity; Changes in connectivity correlated with SWA, delirium severity, interleukin-10, and monocyte chemoattractant protein-1
Thomas et al. (2008) [62]	BMC Neurosci	Prospective	61 (15/46)	86,2	74	Mixed	Mixed collective - dementia considered in analysis	N.m.	DSM/ ICD, CAM, DRS	rEEG	Qualitative & quantitative	10-20 system, 500 Hz sampling rate, 0.03-70 Hz band pass filter, 32 channels, EMG	SAA not associated with delirium or cognitive functions; Occipital slowing, peak power and alpha decrease, delta and theta power increase, and slow wave ratio increase associated with delirium; EEG measures were correlated with cognitive performance and delirium severity, but not SAA
Thomas et al. (2008) [63]	J Neurol Neurosurg Psychiatry	Prospective	50 (12/38)	85,8	72	Mixed	Mixed collective - dementia considered in analysis	N.m.	DSM/ ICD	rEEG	Qualitative & quantitative	10-20 system, 500 Hz sampling rate, 0.03-70 Hz band pass filter, 32 channels, EMG	qEEG was substantially better than rEEG in differentiating patients with delirium and dementia, delirium only, and cognitively unimpaired subjects; Differentiation of patients with delirium vs. with delirium and dementia using qEEG variables activated upper alpha and delta power density with 67% sensitivity and 91% specificity; Differentiation of patients with delirium vs. cognitively unimpaired subjects using qEEG variables relative theta power density at rest with 83% sensitivity and 60% specificity
Trzepacz et al. (1986) [64]	Int J Psychiatry Med	Prospective	40 (12/28)	40	62	Liver Disease	Mixed collective - dementia NOT considered in analysis	Substance abuse reported but NOT considered in analysis	DSM/ ICD	rEEG	Qualitative	N.m.	Delirium associated with serum albumin < 3.0 g/dl, MMSE scores < 24, impairment in TMT-A and -B, EEG dysrhythmia; In a subsample, differentiation between delirious and non-delirious patients using MMSE, TMT-A and -B, EEG, and albumin with 100% specificity and 100% sensitivity
Trzepacz et al. (1988) [65]	Biol Psychiatry	Prospective	108 (18/90)	41	65	Liver Disease	N.m.	Substance abuse reported but NOT considered in analysis	DSM/ ICD	rEEG	Qualitative	10-20 system, 16 or 17 channels	Significantly slower dominant posterior rhythm, lower serum albumin, and worse scores in TMT-A and -B and MMSE in delirious compared to non-delirious patients; Differentiation between delirious and non-delirious patients using TMT-B, EEG, and albumin with 98% specificity and 83% sensitivity
Trzepacz et al. (1989) [66]	J Neuropsychiatry Clin Neurosci	Prospective	46 (23/23)	40,4	60	Liver Disease	N.m.	Substance abuse reported but NOT considered in analysis	DSM/ ICD	rEEG	Quantitative	10-20 system, 120 Hz sampling rate, 4 channels	Significantly worse performance in MMSE, TMT-A and -B, and lower mean peak activity in delirious compared to non-delirious patients; Mean auditory brainstem evoked potentials were abnormal in both groups, with delirious patients showing a bimodal distribution of latency values and a greater proportion of abnormal values; somatosensory evoked potentials were abnormal only for delirious patients
Trzepacz et al. (1989) [66]	Psychosomatics	Prospective	247 (46/201)	41,3	63	Liver Disease	Mixed collective - dementia NOT considered in analysis	Substance abuse reported but NOT considered in analysis	DSM/ ICD	rEEG	Qualitative	10-20 system, 16 or 17 channels	Significantly lower serum albumin, more EEG dysrhythmia, and worse performance in MMSE, TMT-A and -B in delirious compared to non-delirious patients; Delirious patients had significantly poorer adaptive functioning and lower occupational, family, and social scale ratings
Vacas et al. (2016) [67]	Anesth Analg	Prospective	23 (8/15)	68	N.m.	Mixed	N.m.	N.m.	DSM/ ICD, CAM	cEEG	Qualitative	10-20 system, 2500 Hz sampling rate; 0.3-35 Hz band pass filter, 4 channels + eye movement, EMG	Moderate agreement between SedLine and polysomnography monitoring; No differences in delirium occurrence in patients with and without sleep disruption
van Dellen et al. (2014) [68]	Anesthesiology	Prospective	49 (25/24)	75,1	45	Surgery	No subjects with dementia	N.m.	DSM/ ICD, CAM	rEEG	Quantitative	10-20 system, 512 Hz sampling rate, 0.15 Hz high pass filter, 21 channels	Significantly lower mean PLI in the alpha band in delirious compared to non-delirious patients; Network topology in delirious patients characterized by lower normalized weighted shortest path lengths in the alpha band; Significantly lower delta band dPLI in anterior regions and higher in central regions in delirious compared to non-delirious patients
van der Kooi et al. (2015) [35]	Chest	Prospective	56 (28/28)	75,5	43	Surgery	No subjects with dementia	N.m.	DSM/ ICD, CAM	rEEG	Quantitative	10-20 system, 512 Hz sampling rate; 0.5-30 Hz band pass filter, 21 channels + eye movement	Differentiation of delirious vs. non-delirious patients using relative delta power from 1 min artifact-free recording of electrodes F8-Pz with 100% sensitivity and 96% specificity
van Sweden, Mellerio (1989) [69]	Biol Psychiatry	Prospective	16 (16/0)	50,8	69	Intoxication/ Withdrawal	N.m.	Substance abuse reported and considered in analysis	N.m.	rEEG	Qualitative	N.m.	All delirious patients had non-convulsive paroxysmal EEG disturbances without a history of epilepsy

*AUROC* Area under receiver operating characteristic curve, *CAM* Confusion Assessment Methods, *cEEG* Continuous EEG, *DRS* Delirium Rating Scale, *DSM* Diagnostic and Statistical Manual of Mental Disorders, *EEG* Electroencephalography, *EMG* Electromyography, *FT* Fourier transformation, *Hz* Hertz, *ICD* International Classification of Diseases, *ICU* Intensive care unit, *IIR* Infinite impulse response, *LORETA* Low-resolution electromagnetic tomography, *MMSE* Mini Mental State Examination, *N.m.* Not mentioned, *PLI* Phase-lag index, *dPLI* Directed PLI, *rEEG* Routine EEG, *SAA* Serum amyloid A, *SWA* Slow-wave activity, *TMT-A and -B* Trail-Making Test A and B

### Study quality

For an overview of QUADAS-2 quality rating, please see Tables 2 and 3. For detailed ratings, please see supplementary materials. In general, almost all studies had some risk of bias. More specifically, for each of the four domains, the most common issues were A) missing information on sex/gender of patients; B) missing information on EEG data quality and blindness of the rater to delirium diagnosis; C) missing information on time of onset and duration of delirium, as well as confounding factors such as dementia diagnosis, substance abuse, and medication; D) inconsistent timing of EEG and delirium diagnosis. With regard to applicability, there were only minor concerns. The overall inter-rater reliability for the risk of bias assessment was κ = 0.948, and for the applicability assessment κ = 0.778.

**Table 2:** Summary of QUADAS-2-based rating of methodological study quality

		Risk of Bias				Applicability Concerns			
		Patient Selection	Index Test	Reference Standard	Flow & Timing	Patient Selection	Index Test	Reference Standard	
Allahyari et al. (1976) [42]	Psychiatr Clin	✔	✖	**?**	✔	✔	✔	✔	
Azabou et al. (2015) [43]	PLoS One	✔	✔	**?**	✔	✔	**?**	✔	
Evans et al (2017) [44]	Clin Neurophysiol	**?**	✔	✔	✔	**?**	**?**	**?**	
Fleischmann et al. (2019) [45]	Clin EEG and Neurosci	**?**	**?**	✖	✖	✔	✔	✔	
Fleischmann et al. (2019) [45]	Pilot Feasibility Stud	**?**	✔	✖	✖	✔	✔	✔	
Hunter et al. (2020) [46]	AIMS Neurosci	✔	✔	**?**	✔	**?**	✔	✔	
Jacobson et al. (1993) [47]	J Neurol Neurosurg Psychiatry	**?**	**?**	✔	✖	✔	✔	✔	
Jacobson et al. (1993) [47]	Biol Psychiatry	✔	✔	✔	✔	✔	✔	✔	
Katz et al. (1991) [48]	Int Psychogeriatr	**?**	✔	✖	✔	✔	✔	✔	
Keijzer et al. (2020) [49]	Resuscitation	✔	✔	✖	✔	✔	✔	✔	
Kimchi et al. (2019) [50]	Neurology	✔	✔	**?**	✔	✔	✔	✔	
Knauert et al. (2018) [51]	J Crit Care	✖	✔	**?**	✔	✔	✔	✔	
Koponen et al. (1989) [52]	J Neurol Neurosurg Psychiatry	**?**	**?**	✔	✖	✔	✔	✔	
Matsushima et al. (1997) [53]	Biol Psychiatry	**?**	**?**	✔	✔	✔	✔	✔	
Naeije et al. (2014) [54]	Epilepsy Behav	**?**	**?**	✖	✔	✔	✔	✔	
Nielsen et al. (2019)	Neurocritical Care.	✔	✔	✖	✔	**?**	**?**	✔	
Numan et al. (2017) [55]	Clin Neurophysiol		✔	✖	✔	✔	✔	✔	
Numan et al. (2019) [56]	British Journal of Anaesthesia	✔	✔	✖	✔	✔	✔	✔	
Plaschke et al. (2007) [57]	Anaesthesia	✔	✔	✔	✔	✔	✔	✔	
Reischies et al. (2005) [58]	Psychiatry Res	✖	✔	✖	✔	**?**	✔	**?**	
Sambin et al. (2019) [59]	Front Neurol	**?**	**?**	✔	✔	✔	✔	✔	
Sun et al. (2019) [60]	NPJ Digit Med	✔	**?**	**?**	✔	✔	**?**	**?**	
Tanabe et al. (2020) [61]	Br J Anaesth	✔	✔	✖	✔	✔	✔	✔	
Thomas et al. (2008) [62]	BMC Neurosci	✔	✔	**?**	✖	✔	✔	✔	
Thomas et al. (2008) [63]	J Neurol Neurosurg Psychiatry	**?**	✔	**?**	✖	✔	✔	✔	
Trzepacz et al. (1986) [64]	Int J Psychiatry Med	**?**	✖	✖	✖	✔	✔	✔	
Trzepacz et al. (1988) [65]	Biol Psychiatry	✔	**?**	✖	✔	✔	✔	✔	
Trzepacz et al. (1989) [66]	J Neuropsychiatry Clin Neurosci	✔	✔	✖	✖	✔	✔	✔	
Trzepacz et al. (1989) [66]	Psychosomatics	✔	**?**	✖	✖	✔	✔	✔	
Vacas et al. (2016) [67]	Anesth Analg	**?**	✔	✖	✔	✔	✔	✔	
van Dellen et al. (2014) [68]	Anesthesiology	✔	✔	**?**	✔	✔	✔	✔	
van der Kooi et al. (2015) [35]	Chest	✔	✔	**?**	✔	✔	✔	✔	
van Sweden & Mellerio (1989) [69]	Biol Psychiatry	**?**	✖	✖	✔	✔	✔	✔	

✔ indicates low risk of bias and low applicability concerns, **?** indicates unclear risk of bias and applicability concerns due to missing data or mixed quality, ✖ indicates high risk of bias and low applicability concerns

**Table 3:** Summary of inter-rater reliability between raters T.L.T.W. and K.D. in QUADAS-2-based rating of methodological study quality (Cohen’s Kappa)

	Risk of Bias				Applicability Concerns		
	Patient Selection	Index Test	Reference Standard	Flow & Timing	Patient Selection	Index Test	Reference Standard
Cohen’s Kappa	0.967	0.872	0.985	0.954	0.369	1.000	0.841
Total Cohen’s Kappa	0.948				0.778		

### Study and patient characteristics

Among the 33 studies included for analysis, 29 were prospective, and four (12.1%) were retrospective [45, 51, 70, 71]. The studies included an average of 94.6 subjects (range: 10-543), 34.1 patients with delirium (range: 3-129) and 58.0 non-delirious patients (range: 0-414). One study did not specify, which of the patients were delirious [60]. The mean patient age was 66.1 years (standard deviation (SD): 13.7). Three studies did not report age in a way that the mean age could be calculated [42, 48, 60]. 47.1% (SD: 19.2) of patients were female. Two studies did not provide patient sex/gender [48, 67].

Of the 33 studies, six (18.2%) included patients with surgical admission diagnoses [35, 44, 55, 56, 61, 68]; four (12.1%) included patients with liver disease [64–66, 72]; two (6.1%) included patients with alcohol abuse/intoxication [42, 69]; 14 (42.4%) included patients with mixed admission diagnoses [45, 46, 50–52, 57, 59, 60, 62, 63, 67, 70, 71, 73]; in four studies (12.1%), patients had admission diagnoses other than those mentioned [43, 49, 53, 58]; in three studies (9.1%), admission diagnoses were not reported [47, 48, 54].

With regard to confounding factors, eight of 33 studies (24.2%) excluded patients with dementia [35, 44, 46, 50, 51, 57, 68]; six (18.2%) included subjects with dementia and studied differences in EEG between patients with delirium, with dementia, and with both conditions [47, 52, 59, 62, 63, 70]; four (12.1%) included subjects with dementia but did not take this into account in their analyses [45, 56, 64, 72]; 15 (45.5%) did not mention whether subjects with dementia were included [42, 43, 48, 49, 53–55, 58, 61, 65–67, 69, 71, 73]. Furthermore, six studies (18.2%) did not include patients with alcohol/substance abuse [44, 47, 52, 53, 57, 70]; three (9.1%) included patients with alcohol/substance abuse and took this into account in their analysis [42, 59, 69]; six (18.2%) included patients with alcohol/substance abuse but did not consider this in the analysis [43, 56, 64–66, 72]; 18 (54.5%) did not mention whether patients with alcohol/substance abuse were included [35, 45, 46, 48–51, 54, 55, 58, 60–63, 67, 68, 71, 73]. Lastly, one study (3.0%) reported not including patients with medication affecting interpretation of findings [71]; 21 (63.6%) reported medication and considered it in the analysis [35, 42, 43, 46–49, 51–57, 59, 62, 63, 68–71, 73]; four (12.1%) reported medication but did not consider it in the analysis [45, 58, 60, 67]; seven (21.2%) did not mention medication [44, 50, 61, 64–66, 72].

Eighteen (54.5%) of the 33 included studies used DSM-3/4/5 or ICD-10 criteria for diagnosing delirium [35, 47, 49, 52, 53, 55, 56, 58, 62–68, 70–72]; 16 (48.5%) used variations of the CAM for diagnosing delirium [35, 43–46, 50, 54, 56, 57, 59–61, 63, 67, 68, 73]; four (12.1%) used variations of the DRS for diagnosing delirium [44, 56, 58, 61]; one study (3.0%) used another chart based method [51]; and in three studies (9.1%), no tool for diagnosing delirium was reported [42, 48, 69].

Cerebral imaging was used in seven studies (20.6%) [52, 53, 59, 61, 62, 69, 71]. One of them showed impaired structural connectivity in diffusion tensor imaging (DTI) [61]. Eleven studies (33.3%) collected blood samples in their protocol [43, 53, 54, 57, 59, 61, 63–65, 69, 72]. The findings varied and abnormalities were often mild. Of note, two studies found serum anticholinergic activity, a possible blood biomarker of delirium [57], not to be associated with delirium [57, 63]. In three studies by Trzepacz et al. on delirium due to liver disease, serum albumin was significantly decreased [64, 65, 72].

### EEG

#### Technical aspects

The recordings and analyses of EEGs varied greatly between the studies. There were major differences in positioning and number of electrodes, type of montages, duration of recording, and evaluation methods of EEG data. For details, please see supplementary materials.

#### Continuous EEG vs. routine EEG

Most studies (23/33) performed rEEG recordings, with a duration of 20 to 30 min. Numan et al. [56] performed sequential five-minute recordings, one prior to surgery and one recording for each of the first 3 days after surgery. Eight studies performed cEEG recordings with a mean recording time of 19 to 44 h [44, 48, 49, 51, 55, 59, 67, 73] (one study did not specify duration of recording [55]). Naeije et al. [54] compared the sensitivity of rEEG vs. cEEG with regard to detection of epileptic discharges or non-convulsive status epilepticus (NCSE) in association with delirium and therefore used both, rEEGs and cEEGs.

#### Qualitative and Quantitative analysis methods

##### Qualitative

Six studies analyzed EEGs by using qualitative and quantitative methods [47, 53, 62, 63, 70, 73]. Eleven studies analyzed EEGs by only using qualitative methods [42, 43, 50, 51, 54, 59, 64, 65, 67, 69, 72]. Only three of these used a standardized classification system, i.e., the Mayo Clinic classification system [64, 65, 72]. Knauert et al. [51] and Azabou et al. [43] used an encephalopathy classification introduced by Synek et al. [74] in 1990. Azabou et al. [43] also used the Young classification [75]. Three studies used qualitative methods also for cEEG [51, 54, 67]. Sambin et al. [59] and Naeije et al. [54] searched for characteristic patterns of epileptic activity without describing alternative findings. Vacas et al. [67] used qualitative methods to label different sleep phases and quantify the amount of time spent in each phase. Allahyari et al. [42] examined patients with delirium tremens. They attribute slow waves to effects of medication and classified EEGs as either normal or abnormal. Only exemplary cases were presented in detail. Sweden and Mellerio [69] analyzed qualitative aspects of EEGs recorded during drug withdrawal states in patients with signs of delirium but also clinical signs of epilepsy. Therefore, EEG findings focus on typical epileptic discharges. Finally, Kimchi et al. [50] performed a thorough qualitative analysis and description of rEEGs. The six studies that combined qualitative and quantitative analysis used, for the qualitative part, similar methods as the ones mentioned above. Two used the Mayo Clinic classification system [47, 70]. Matsushima et al. [53] only described whether EEGs were classified as normal or showed a degree of slower activity. The other three studies [62, 63, 73] performed a thorough qualitative analysis. Of these, Thomas et al. [62] also quantified rate of reactivity, frequency variation, and delta excess.

##### Quantitative

Most studies (21/22) performing quantitative analyses of rEEGs or cEEGs used a frequency domain-based method that subdivides complex waveforms in specific frequency components by using Fast Fourier Transformation (FFT). The remaining one used a time domain-based method of waveform analysis that independently measures amplitude and duration of each wave, in order to detect changes that affect only one of these two components [53]. For technical aspects of FFT and waveform recognition, please see supplementary materials.

#### EEG findings in delirium

##### Qualitative

The most common findings in qualitative EEG analysis of delirious patients were occipital slowing, excess delta or theta, anteriorization, and loss of reactivity [43, 59, 62, 63, 65, 73]. In the studies by Knauert et al. [51] and Azabou et al. [43], most patients were diagnosed with moderate, moderate to severe, or severe encephalopathy.

##### Quantitative

With regard to choice of electrode derivations, most studies used the whole range of electrodes and compared each electrode to a reference. Others averaged electrodes of anatomical regions to search for differences. Six studies [35, 45, 46, 52, 53, 56] used two- or three-electrode derivations. More specifically, Hunter et al. [46], van der Kooi et al. [35], and Fleischmann et al. [45] examined different derivations among all included electrodes, while Koponen et al. [52], Matsushima et al. [53], and Numan et al. [56] just analyzed the T6-O2 or T5-O1 derivation, the C3-O1 derivation, or Fp2-Pz and T8-Pz derivations, respectively.

Hunter et al. [46] generated a ratio between slow (< 13 Hz) and fast (13-45 Hz) frequencies derived from the electrodes C3, P3 and T7. Van der Kooi et al. [35] evaluated quantitative EEG data of bipolar electrode derivations. They studied patients under two conditions to identify which setting achieves the highest accuracy in delirium detection: A) ICU patients with eyes open (15 derivations, since all frontal, temporal and parietal electrodes were excluded to avoid blinking artefacts), and B) ICU patients with their eyes closed (210 derivations from 21 electrodes: F10, F9, Fp2, Fp1, F8, F4, Fz, F3, F7, T8, C4, Cz, C3, T7, P8, P4, Pz, P3, P7, O2, O1). They showed that 60 s artifact-free EEG recordings in ICU patients with closed eyes could discriminate delirious from non-delirious patients by just using two electrodes. An increase in relative delta power of delirious patients in derivations P8-Pz and Fp2-Fpz showed the highest sensitivity (100%) and specificity (95-96%). Fleischmann et al. [45] did a similar in-depth analysis for each of the 210 derivations × 70 frequencies. They identified F3-C4, F3-P4, and O2-F3 at 2 Hz as best classifiers to distinguish patients with and without delirium. These results were confirmed when tested on an unmatched large sample of controls with normal EEGs and an even larger real-world population. Applied to the latter, F3-C4 and F3-P4 at 2 Hz achieved sensitivities of 100% and specificities of 91 and 93%, respectively. When combined with C3-O1 at 19 Hz, specificity increased to 95%. Thus, all three studies demonstrate high sensitivities and specificities for derivations with increased relative delta band in frontal and parieto-occipital regions.

Matsushima et al. [53] found similar results by positioning the two electrodes only in central and occipital regions. In their study the theta/alpha ratio was increased even prior to clinical delirium manifestations. However, this result must be interpreted with caution due to the small sample size (*n* = 20). In addition to the above-mentioned studies included in this review, researchers around Gen Shinozaki have applied the novel bispectral EEG to delirium [76–79]. They showed that using two electrodes only, algorithms based on quantitative EEG can differentiate between delirious and non-delirious individuals as well as estimate prognosis and mortality of delirious patients [76–78]. Furthermore, the bispectral EEG device shows benefits with regard to small size and simple application. Of note, these articles report only modified and pre-interpreted EEG signals and were thus excluded during the literature search.

Koponen et al. [52] also found significant results for a reduced delta frequency in the P7-O1 or P8-O2 derivations. Since other electrode combinations were not derived, a direct comparison with the above-mentioned results is not possible. Moreover, a number of the elderly patients included in the study by Koponen et al. [52] showed substantive cognitive decline, which may explain a proportion of EEG results [62, 70] affecting specificity for delirium detection. Numan et al. [56] found a significant increase in delta power (frequency 0-4 Hz or 0-6 Hz) of delirious patients by using Fp2-Fz and T8-Fz derivations.

With regard to the lower frequencies, most (17/22) quantitative studies showed an increase in relative and absolute power in spectral analysis in delta and theta (mostly in frontal regions), and a decreased relative and absolute power in alpha (mostly in occipital or parietal regions) in delirious compared to non-delirious patients [35, 45, 46, 48, 49, 52, 55–58, 61–63, 68, 70, 71, 73].

With regard to the higher frequencies, Fleischmann et al. [45] also highlighted the importance of a decrease in the relative beta power in detecting delirium, especially in the C3-O1 derivation. Nielsen et al. [73] and Hunter et al. [46] also observed a reduction of beta activity in qualitative analysis of EEGs recorded from delirious ICU patients. In the study by Numan et al. [56], a decrease in relative beta power was one of the best discriminators for delirium detection, as shown by a random forest classifier. Hunter et al. [46] report a substantial reduction of gamma power in five delirious compared to five non-delirious patients. However, there were no differences in gamma power in the much larger study by Fleischmann et al. [71]. Tanabe et al. [61] also observed a decrease in high frequencies among delirious patients.

Moreover, one study reports a decreased centroid frequency (i.e., frequency that divides area of the spectrum in two equal parts) [57], another a decreased peak frequency [52], and two studies a decreased mean frequency [52, 66]. Other parameters described are increased theta/alpha ratio [53], decreased alpha/theta ratio [52, 57], decreased (alpha + beta)/(theta + delta) ratio [52], and a decreased scaled alpha-to-delta ratio, defined as the ratio of EEG power in the alpha band and delta band [49]. Keijzer et al. [49] also looked at the fraction of time not spent in suppression in EEG, which was lower in delirious patients after cardiac arrest compared to non-delirious patients.

With regard to connectivity analyses, the studies by Numan et al. [55], van Dellen et al. [68], and Tanabe et al. [61] found a significantly lower average phase lag index (PLI) for the alpha frequency band in delirious compared to non-delirious patients.

The study by Numan et al. [55] also found loss of posterior-anterior directionality in the alpha band, and loss of integration of the network organization. The latter was shown by the comparison of minimum spanning tree (MST) measures between hypoactive delirium patients and non-delirious patients. Delirious patients showed a decrease in degree, leaf fraction, and maximum betweenness centrality in the alpha band during delirium. Similarly, also Numan et al. [55] found a disturbed posterior-anterior connectivity in the alpha band. Van Dellen et al. [68] also report a decreased path length in the alpha band of delirious patients compared to controls. On the other hand, the clustering coefficient and small-world index did not differ between the groups. With regard to directed connectivity, delirious patients in both studies demonstrated a loss of posterior to anterior orientation in the alpha band [55, 68]. In the study by van Dellen et al. [68], patients also showed a lower delta band directed PLI (dPLI) in anterior regions and a higher dPLI in central regions than non-delirious patients [68]. This may indicate a flow of information within the delta band towards frontal regions. There were no differences in posterior regions or in dPLIs of other bands [68]. Of note, Tanabe et al. [61] found an increased frontal functional connectivity in patients that developed a postoperative delirium. The authors hypothesize a compensatory mechanism for a decreased structural connectivity (most likely due to neurodegenerative processes). Impaired structural connectivity has been confirmed by DTI studies [61, 80].

Fleischmann et al. [71] found global alpha and regional beta band disconnectivity as well as theta band hyperconnectivity in delirious patients. Similarly, also Hunter et al. [46] reported a general disconnectivity in delirious patients. A link between disturbance of consciousness and disconnectivity in the alpha band has already been shown in studies on the effects of ketamine [81] and propofol [82]. Thus, Fleischmann et al. interpret their findings as a sign of disturbed consciousness in delirium. In fact, the abnormalities in connectivity were spread across multiple networks engaged in consciousness, attention, working memory, executive functioning, and salience detection. In summary, functional connectivity seems to be impaired in delirious patients. Differences in affected regions, direction of connectivity, and affected band ranges might be explained by small sample sizes, divergent patient populations, different methods and study designs.

##### Discrimination of different types of delirium

Spectral EEG analysis of 51 ICU patients with hyperactive, hypoactive, and mixed types of delirium did not demonstrate any significant difference in relative alpha, beta, theta, or delta power, alpha/theta ratio, (alpha + beta)/(theta + delta) ratio, or mean frequency values between the different delirium types [52]. This result is limited by the small number of patients in the different categories and the fact that most patients were diagnosed with dementia, which may explain some of the EEG changes. In line with these results, Numan et al. [56] did not find a significant difference in relative delta power between different types of delirium by using three electrodes. Van Dellen et al. [68] also studied delirious patients with and without hallucinations. Presence of hallucinations did not make any difference in alpha band PLI values, path length in graph theoretical analysis, or anterior to posterior dPLI gradient. However, patients with hallucinations showed a significant lower clustering coefficient and small-world index compared to delirious patients without hallucinations.

##### Severity of delirium and outcome

Tanabe et al. [61] reported a high correlation between an increase of slow wave activity in occipital regions and deliriums severity. The highest correlation was found at electrode Oz. In the study of Knauert et al. [51], reduction or absence of K-complexes during delirium was associated with worse outcome. Moreover, absence of sleep spindles correlated with unfavorable modified Rankin Scale scores.

##### EEG changes in delirious patients over time

Matsushima et al. [53] reported a significant slowing in recordings from central and occipital electrodes prior to a clinically overt delirium. This was measured by the theta/alpha ratio derived by a quantitative waveform recognition method of individuals in a serial measurement over many days after surgery. As mentioned above, Tanabe et al. [61] observed an increased alpha power preoperatively in patients who developed delirium postoperatively. Nielsen et al. [73] found continuous delta or theta activity in cEEG, loss of beta activity, and reactivity in evolving delirium. Resolution of delirium was characterized by re-occurrence of beta and reduction in delta activity. In an early study by Jacobson et al. [47], delirious patients that improved in cognitive functioning up to 19 months after the initial testing showed a significant increase in relative alpha band power and brain map changes with reduction of theta and delta in follow-up EEGs. However, the results are limited by a small sample size (*n* = 34), selection bias, a number of confounders, and absence of reporting of a follow-up delirium score associated with the second EEG.

##### Sleep patterns

Through analysis of sleep patterns in EEG, Evans et al. [44] demonstrated that patients with delirium following routine surgery need more time to fall asleep and sleep less during the first night after surgery prior to presenting clinical signs of delirium. In addition, delirium severity was negatively correlated with the amount of sleep during this first night as well as latency to falling asleep. Vacas et al. [67] did not find any difference in polysomnographic variables in cEEG between ICU patients that developed delirium and those that did not. Results are limited by a small sample size (*n* = 23), missing temporal and occipital electrodes, and duration and time of EEG (only assessed the first day after surgery). Despite the limitations of both studies, loss of physiological sleep structure may be an early indicator of delirium. This is in line with a previous study that used actigraphy [83]. EEG seems to offer a benefit since it provides objective criteria for sleep. Furthermore, the validity of estimates of latency of sleep onset and total duration of sleep based on self-reports is limited [84]. Further research is needed to evaluate the role of EEG, time point of recording, number of electrodes, and type of analysis as an early predictor for the development of delirium.

##### Epileptic activity

Using 24 h cEEG, Sambin et al. [59] identified sporadic epileptiform discharges (SEDs) in ten of 50 (20%) delirious patients. Periodic discharges (PDs) were observed in eleven of 50 patients (22%), eight of which had generalized PDs (GPDs), and three had lateralized PDs (LPDs). Moreover, seven of 50 patients (14%) had seizures, six of which had NCSE. Similarly, in the study by Azabou et al. [43], five of 22 (23%) delirious and six of 42 (14%) non-delirious patients had PDs. In addition, seven of 22 (32%) delirious and four of 42 (10%) non-delirious patients had electrographic seizures. Naeije et al. [54] found NCSE in cEEGs of nine of 32 (28%) delirious patients in the emergency department. rEEG detected NCSE only in two (6%) of the 32 patients. Nielsen et al. [73] observed no evidence of NCSE in cEEG recordings of delirious patients. However, they detected lateralized or bilateral PDs in 13 of 66 (20%) delirious, and two of 36 (6%) non-delirious patients.

Although delirium has shown to be associated with ictal or post-ictal conditions [85], the role of interictal activity is less clear. The incidence of SEDs, LPDs, GPDs, or NCSE in delirium cannot be calculated by these studies, due to the small number of patients, selection bias and bias through concomitant treatments with antiepileptic medication. However, cEEG in this setting can improve the diagnostic performance.

##### Discrimination of delirium from other conditions

Jacobson et al. [70] discriminated patients with dementia, delirium, both conditions, or no encephalopathy. More specifically, normal from encephalopathic records could be differentiated by Mini-Mental State Examination (MMSE) in 85% of cases, by relative power in alpha in 91%, and MMSE plus relative power in alpha in 94% of cases. Delirium and dementia could be differentiated by theta activity (89%), brain map rating (89%), or combinations of theta activity, brain map rating, and/or relative delta power (up to 93%). Similar results were achieved by Koponen et al. [52]. Based on relative delta/alpha2-power density during activation in qEEG, Thomas et al. [62] differentiated patients with dementia and delirium from patients with delirium in 83% of cases. Numan et al. [55] demonstrated a less integrated and less organized functional network of delirious patients compared to healthy controls as shown by the reduced degree, leaf fraction, and maximum betweenness centrality in the alpha band. However, similar results were obtained in patients with Lewy Body Dementia and Alzheimer’s Disease, limiting the specificity [86, 87]. A direct comparison between patients with delirium and patients with dementia using advanced network analysis methods with more electrodes is needed to evaluate the discriminatory potentials of EEG in this context.

In the study by Numan et al. [55], both delirious patients as well as patients recovering from anesthesia (both with altered consciousness) showed an increased relative delta power and reduced relative alpha power in spectral analysis. Although the reduction of relative alpha power was stronger among patients with hypoactive delirium, the discriminatory potential of a spectral analysis between delirious patients and patients during recovery from anesthesia was low. This was also the case when applying functional connectivity measures such as PLI and directionality of connectivity [55]. Neuroleptic drugs were previously said to influence EEG [88, 89]. However, van der Kooi et al. [35] and Koponen et al. [52] did not find differences in relative delta power reduction in delirious patients that were treated with either haloperidol or chlorpromazine-equivalents, compared to delirious patients that were not treated. Other medication that could influence the EEG were often not reported systematically.

##### Sensitivity and specificity

Qualitative EEG parameters cannot effectively discriminate patients with delirium and patients with dementia [53, 62]. In contrast, quantitative methods such as computerized waveform analysis demonstrated a significant difference in the ratio of theta over alpha waves [53]. In addition, other quantitative parameters like the increase of the relative delta band or reduction of the relative alpha band showed much higher odds ratios and reached 67% sensitivity and 91% specificity in one study [62]. In two studies by Trzepacz et al. [64, 65], a qualitative EEG analysis of delirious patients after liver transplantation demonstrated sensitivities of 83 and 75%, and specificities of 78 and 88%, respectively, for reduced dominant posterior rhythm. Both studies are limited by small sample sizes (*n* = 12 and 18 delirious patients) and selection bias.

In two studies, an increase of relative theta power in quantitative analysis was identified as the most sensitive characteristic of delirious patients compared to non-delirious patients [62, 63]. Numan et al. [55] performed a random forest analysis including spectral analysis, functional and directed connectivity and network topology. 77% sensitivity and 95% specificity were reached. Relative alpha, beta, and delta powers were the best discriminators. Elaborate quantitative analysis of > 200 bipolar derivations in all frequency bands did not confirm the expected sensitivity of the relative power in the theta band [35]. One reason for this could be the different margins of the theta frequency. Thomas et al. [62, 63] sub-classified the theta frequency into a lower frequency part (3-5 Hz) and an upper frequency part (5-7 Hz). The high sensitivity applied only to the lower part, which is nearer to the delta frequency that showed the highest sensitivity in the study by van der Kooi et al. [35]. Moreover, Numan et al. [56] found a better discriminatory potential between delirious and non-delirious patients in a 1-6 Hz frequency range than 1-4 Hz. Van der Kooi et al. [35] achieved around 100% sensitivity and 95% specificity for certain bi-electrode derivations, as mentioned above. Fleischmann et al. [45] found similar sensitivity and specificity in a larger not pre-specified cohort. Still, there are limitations to both studies [35, 45] since the sensitivity of the confirmation test (i.e., CAM-ICU) based on which patients were divided into delirium and non-delirium groups has been shown to be only 47% in the routine clinical setting [31]. Finally, EEG was performed only once. Thus, longitudinal studies on non-pre-selected groups of patients with quantitative cEEG are needed to further investigate sensitivity and specificity of EEG in delirium detection.

## Discussion

### Summary

EEG seems to offer manifold possibilities in diagnosing delirium. All studies showed a certain degree of qualitative or quantitative EEG alterations in delirium. Thus, normal rEEG and cEEG make presence of delirium very unlikely. Also, some studies have pointed toward the potential of EEG to differentiate delirium from other disorders such as dementia. However, included studies yielded only limited insights as to how EEG may help differentiate different types of delirium and underlying etiologies. Moreover, only a few studies investigated EEG findings of patients prior to developing delirium. Thus, it remains unclear whether EEG may help predict what patients ultimately develop delirium. Further studies using quantitative EEG methods in thoroughly characterized patient populations are needed to find elements that could identify patients at risk for delirium. To further study brain abnormalities underlying delirium, EEG should be combined more often with advanced neuroimaging such as DTI or functional magnetic resonance imaging. Few studies have pointed toward loss of physiological sleep structure as a potential early indicator of delirium which should be further investigated. In addition, there were mixed findings with regard to the value of serum anticholinergic activity as blood biomarker for delirium.

Although most studies used slightly different methods, spectral analysis seems to be a promising method in identifying delirium. An increase in delta power in frontal, central or temporal regions alone, or in combination with a reduction in beta frequencies in occipital regions measured by only two electrode derivations showed a high sensitivity and specificity. These findings could lead to development of simple diagnostic algorithms that could help to early identify ICU patients at risk for delirium. In addition, the knowledge gained could be used to improve other EEG-based methods, such as bispectral index algorithms, which have recently shown promising results [78, 79, 90–92]. We could find only single studies reporting EEG findings that could help in prediction of duration or severity of delirium. Similarly, almost no studies used EEG to predict patient outcome.

Even though studies included in this review are not sufficient to determine the exact incidence or role of epileptic potentials in delirium, the number of patients with these findings is striking. Thus, 24-48 h cEEG monitoring in patients with delirium may be of great value. Moreover, studies testing treatment strategies for NCSE as well as for interictal epileptic activity, LPDs, or GLDs should be considered.

### Limitations

As a major current limitation, all 33 studies used different research protocols to at least some extent. These include differences in time points, duration, conditions, and recording methods of EEG, as well as different patient populations, and diagnostic methods. In addition, many studies did not adequately control for effects of alcohol/substance abuse or medication. To identify EEG signals that are specific to delirium, studies on patients excluding the effects of medication or other confounding substances as well as comorbidities are needed. Further, regarding study quality, missing information on sex/gender of patients, on EEG data quality and blindness of raters, on time of onset and duration of delirium, and inconsistent timing of EEG were common. One further limitation is the segregation of published literature with regard to encephalopathy and delirium. In a recent paper [93], a consensus on the nomenclature of delirium and encephalopathy based on a statement of ten societies was reached. Nevertheless, in the older literature, different terms like “acute confusional state”, “acute brain dysfunction”, or acute “altered mental status” are commonly used and could mask delirium. We focused on articles that explicitly diagnosed delirium in order to avoid bias. Lastly, there may be a considerable patient overlap in several of the included studies.

### Conclusion

Proposals for unified diagnostic approaches and subsequent prospective studies in non-pre-selected patient cohorts with commonly used, well documented, and standardized delirium-assessments are necessary to calculate sensitivity and specificity. Thus, a quantitative synthesis and common recommendations are so far elusive. Future studies should compare the different methods of EEG recording and evaluation to identify robust parameters for everyday use. Evidence for quantitative bi-electrode delirium detection based on increased relative delta power and decreased beta power is growing and should be further pursued. Additionally, the evolution of a delirium has rarely been addressed so far. Future studies should associate EEG-based biomarkers of delirium with patient outcomes.

## Supplementary Information

**Additional file 1.**

## Data Availability

All data generated or analyzed during this study are included in this published article and its supplementary information files.
