# Supplementary material for: Electroencephalography in delirium assessment: a scoping review
Source: BMC Neurol. 2022 Mar 11;22:86. doi: 10.1186/s12883-022-02557-w (PMC8915483; doi:10.1186/s12883-022-02557-w)
Supplement: Supplementary file 1 — Additional file 1. [file 12883_2022_2557_MOESM1_ESM.docx]

**Supplementary Materials**

***Comprehensive Search Strategy***

Databases: PubMed, EMBASE

*PubMed*:

(((delirium[MeSH Terms]) OR (alcohol withdrawal delirium[MeSH Terms]) OR (delirium of mixed origin[MeSH Terms]) OR (Delirium) OR (Encephalopathy[Title/Abstract]))

AND

((eeg[MeSH Terms]) OR (EEG) OR (Electroencephalography)))

*Embase*:

(Electroencephalography monitoring/ or electroencephalography/ or electroencephalographyphase synchronization)
And
(delirium/ or alcoholic delirium/ or postoperative delirium/ or delirium tremens/ or encephalopahty)

***Detailed QUADAS-2 Risk of Bias Rating***

***
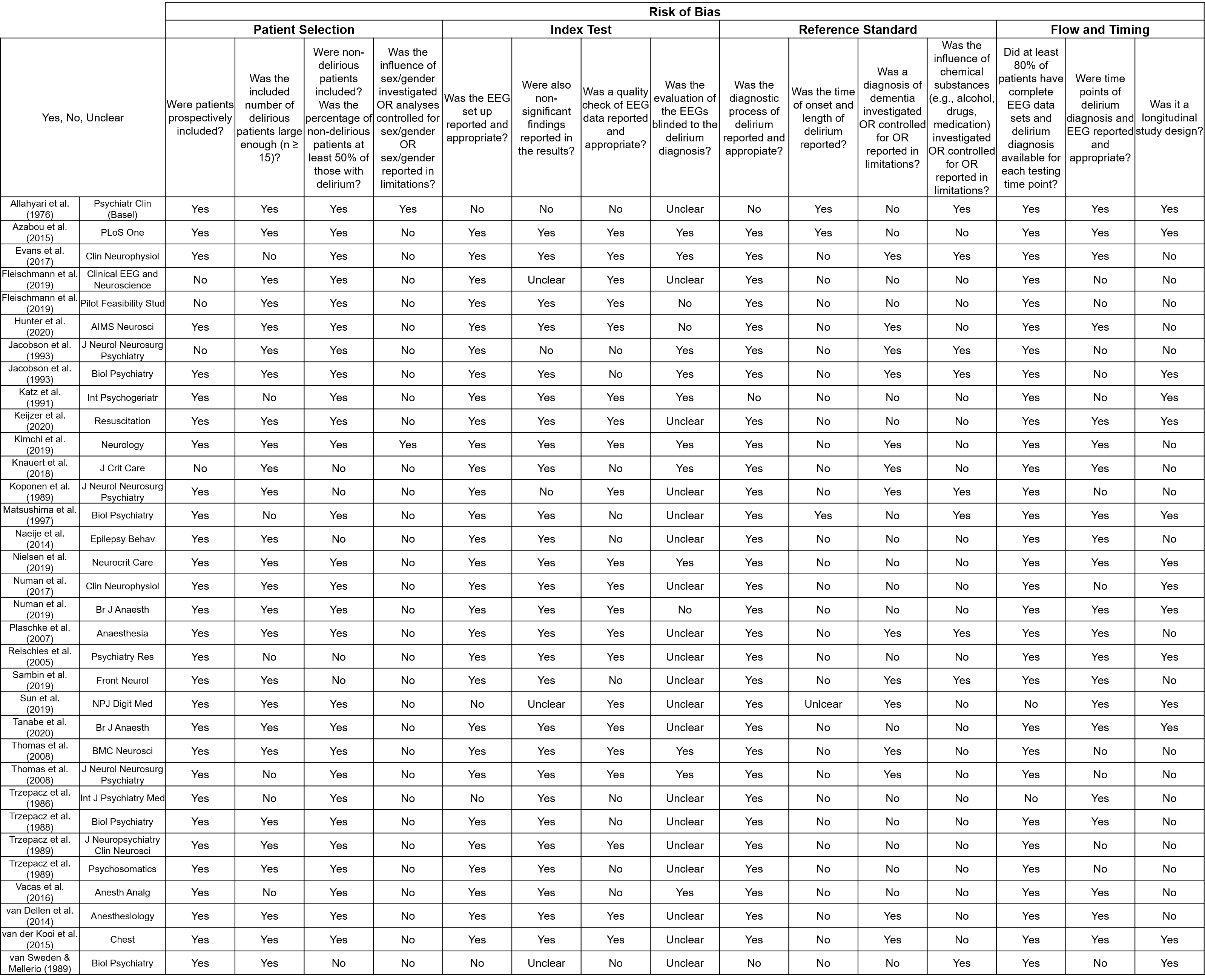
***

***Technical Aspects***

*Montages and Electrodes:*

Of the 33 studies included, 28 used the international 10/20 electrode placement system. The others did not specify placement of electrodes [1-5]. Seven studies did not mention the number of electrodes used [1-3,5-8]. One study analyzed electroencephalography (EEG) recordings using only three electrodes (i.e., FP2, T8 and Pz) [9]. Four studies used four electrodes: Matsushima et al. [10] only used a frontal (Fp1), a central (C3), and an occipital (O1) electrode with a left ear reference electrode for evaluating slow wave proportions in a qualitative and quantitative way; Vacas et al. [11] performed a continuous analysis of sleep potentials using the Fp1, Fp2, F7, and F8 electrodes; Trzepacz et al. [12] used the O1,O2, C3, and C4 electrodes to record routine EEGs (rEEGs) and perform quantitative analysis; Katz et al. [13] used parietal and occipital electrodes (P2-O2, P3-O1) to quantify the background rhythm. Twenty studies used between 16 and 32 electrodes for recording rEEGs or continuous EEGs (cEEGs). Fourteen studies did not specify use of reference electrodes. Two studies referenced to a common average [4,14]. The remaining 17 studies used Cz, Fpz, C2, C3/C4, or A1/A2 (mostly to the ipsilateral ear, but one study explicitly referred to the left ear [10], and another one to the contralateral ear [1]). In addition, six studies used EMG electrodes [1,11,15-18], and eight added electrodes to track eye movements [10,11,17-21]. Moreover, half of the studies (16/33) did not specify the sample frequency, or other recording settings like filters (e.g., filter frequency boundaries or usage of a notch filter). Most recordings used a sampling rate between 250 Hz and 1000 Hz. Low pass filters were mostly set to 70 Hz (range: 35 to 70 Hz) and high pass filters to 0.5 Hz (range: 0.03 to 1 Hz). One study also used second order filters, such as the Butterworth filter [22]. Comparability between studies is further limited by the different margins authors defined for frequency bands. Most used the conventional classification: delta (0.5-4 Hz), theta (4-8 Hz), alpha (8-13 Hz), and beta (13-20 Hz), but many others varied the upper or lower margins, or added sub-classifications in the alpha or theta frequency.

*Technical Aspects of Fast Fourier Transformation:*

Before applying Fast Fourier Transformation (FFT), EEG signals need to be processed. Some studies provide details on digital signal processing. For example, Evans et al. [1] performed an autoregressive high-pass filtering, Plaschke et al. [23] a zero-phase high-pass filtering, and Numan et al. [9] used a 50 Hz notch filter. With regard to the offline re-referencing process, Plaschke et al. [23], Thomas et al. [15,16], van Dellen et al. [24], and Fleischmann et al. [14] used the common average (with 16 electrodes or 32 electrodes). The rest of the included studies do not mention this step. Evans et al. [1], Plaschke et al. [23], Koponen et al. [25], Keijzer et al. [26], and Fleischmann et al. [7] describe the method they used to design a finite impulse response (FIR) filter. All of them used the windowing method by applying a Hamming window. Fleischmann et al. [14] used a multitaper FFT based on discrete prolate spheroidal sequences windowing. Van der Kooi et al. [27] and Tanabe et al. [4] only mention using a FIR filter with cut-off frequencies of 0.5 and 30 Hz, and 0.1 to 50 Hz, respectively. Numan et al. [9] combined FIR with infinite impulse response filtering to correct for differences in the EEG signal and allow comparability with previous studies. The number and duration of epochs used as well as the criteria for their selection varied among studies. Duration ranged between 2 and 120 seconds (2 seconds [1,15,16,21]; 4 seconds [19,20,23]; 8 seconds [22,24,25]; 60 seconds [9,27]; 120 seconds[17]), and number of epochs between 20 and 300. Some authors chose to randomly select epochs out of the whole EEG recording [21]. Others defined a specific time point with high likelihood to be free of artefacts [15,16], or the first artefact-free interval was selected [27]. To avoid artefacts, most studies performed a visual quality check of the selected epochs, excluded frontal or auricular electrodes [14,24,27] (that are known to be susceptible for blinking artefacts), filtered out epochs with sleep stages, and/or excluded high frequencies (>30 Hz) to minimize impact of muscle artefacts [24]. Moreover, a semiautomatic artefact reduction was used in some studies study [4,14,15].

To obtain spectra densities, three studies applied the Welch’s method [4,22,26]. Numan et al. [22], Koponen et al. [25], and Keijzer et al. [26] selected windows of 2-4 seconds length with a 50% overlap. Keijzer et al. [26] additionally applied a Hamming window. Plaschke et al. [23] mention an overlap of 0.4 seconds. Finally, spectral power estimates were mostly averaged per electrode [1,22,25,27]. Some authors averaged over anatomical regions. For example, Thomas et al. [15,16] defined parieto-occipital electrodes (O1, O2, O9, O10, P7, P8, Pz, P3, P4) to generate one value for this whole region. Tanabe et al. [4] performed a log10 transformation to normalize the distribution. Fleischmann et al. [14] and Hunter et al. [28] standardized power bands of each individual by their mean power, in order to facilitate comparability between subjects and studies.

*Waveform Recognition Method*:

Matsushima et al. [10] used 30 second intervals for the computer analysis of the waveform. Output was limited to the ratio of theta (percentage over time) to alpha waves.

*Localization:*

Brain maps were created by calculating values for each electrode that were presented on a two-dimensional colored brain graph [19,20]. Scale maxima used in different studies ranged between 103 to 215 µV^2^ for absolute powers, and 75% to 100% for relative powers [19,20]. Some authors [19,20] developed a score with which they presented the outcome of brain map analysis. This score was derived by adding up a sub-score for delta (0: no excess delta, 1: delta < alpha, 2: delta = alpha, 3: delta > alpha, 4: delta > alpha and delta > theta) and one for theta (0: no excess theta, 1: theta < alpha, 2: theta = alpha, 3: theta > alpha). The results of this score were used for further statistical analysis.

Reischies et al. [21] performed a low-resolution electromagnetic tomography analysis (LORETA) source analysis to investigate intracerebral distribution of electrical activity.

*Network Analysis*:

Numan et al. [22], van Dellen et al. [24], and Tanabe et al. [4] performed a functional connectivity analysis by using the phase lag index (PLI) as a measure of synchronization and, thus, strength of connectivity. Fleischmann et al. [14] used a weighted PLI (wPLI), which weights phase lags and leads with the magnitude of the imaginary component of the cross-spectrum. Hunter et al. [28] used a renormalized partial directed coherence (rPDC) method to estimate causal relationships between regions and, thus, estimate functional connectivity. While Numan et al. [22] re-referenced towards a source reference, van Dellen et al. [24] and Tanabe et al. [4] used an average reference (Tanabe et al. [4] averaged PLI among 25 defined squares, in which they had divided the 256 electrodes). Numan et al. [22] and van Dellen et al. [24] compared an average over all PLI values per frequency band of all channel pairs between the different patient groups. Tanabe et al. [4] performed pairwise analyses between the PLI of the above-mentioned squares. For estimation of the direction of information flow, van Dellen et al. [24] used the directed PLI (dPLI). Numan et al. [22] instead used the phase transfer entropy (PTE) method. dPLI has two major disadvantages: Direction of causal influence is not clear, and results can be influenced by the EEG reference [22]. In order to calculate an anterior-posterior ratio, Numan et al. [22] compared PTE values between frontal and posterior regions. PTE values of frontal electrodes (F7, F3, F4, F8, Fz) were averaged and divided by values of posterior electrodes (i.e., occipital: O1, O2, temporal: T5, T6, and parietal: P3, P4, Pz). Van Dellen et al. [24] used dPLI values of anterior, central, and occipital regions (i.e. anterior: F3, F4, F7, F8, Fz, central: C3, C4, T7, T8, Cz, and posterior: P3, P4, P7, P8, Pz, O1, O2). Sub-networks with maximum connectivity were analyzed using the minimum spanning tree (MST) method [22] for all different bands. MST network measures were then compared among the different patient groups.

To quantify connectivity, van Dellen et al. [24] and Fleischmann et al. [14] performed a graph theoretical analysis based on a method described by Watts and Strogatz [29]. Main parameters defined by this method are the clustering coefficient, that describes the interconnectedness of nodes, and the shortest path length, a measure for network integration. These parameters can be used to assess the small-worldness of the network; the definition of a small-world networks is a high clustering coefficient and short path lengths. Van Dellen et al. [24] only calculated the here-mentioned parameters for bands with significant difference in functional connectivity measures. The graph theoretical analysis approach has been criticized as not adequate for quantifying network structures [30]. Fleischmann et al. [14] divided the functional map into parcels based on data of the human connectome project and calculated the largest eigenvector of centrality parameters. Finally, they compared data with functional magnetic resonance imaging (fMRI) data of previous studies.

**References**

1. Evans JL, Nadler JW, Preud'homme XA, Fang E, Daughtry RL, Chapman JB, Attarian D, Wellman S, Krystal AD (2017) Pilot prospective study of post-surgery sleep and EEG predictors of post-operative delirium. Clinical neurophysiology : official journal of the International Federation of Clinical Neurophysiology 128 (8):1421-1425. doi:10.1016/j.clinph.2017.05.004

2. Trzepacz PT, Maue FR, Coffman G, Van Thiel DH (1986) Neuropsychiatric assessment of liver transplantation candidates: delirium and other psychiatric disorders. International journal of psychiatry in medicine 16 (2):101-111. doi:10.2190/h39m-5utc-hy87-bpx9

3. van Sweden B, Mellerio F (1989) Toxic ictal delirium. Biological psychiatry 25 (4):449-458. doi:10.1016/0006-3223(89)90198-4

4. Tanabe S, Mohanty R, Lindroth H, Casey C, Ballweg T, Farahbakhsh Z, Krause B, Prabhakaran V, Banks MI, Sanders RD (2020) Cohort study into the neural correlates of postoperative delirium: the role of connectivity and slow-wave activity. Br J Anaesth 125 (1):55-66. doi:10.1016/j.bja.2020.02.027

5. Sun H, Kimchi E, Akeju O, Nagaraj SB, McClain LM, Zhou DW, Boyle E, Zheng WL, Ge W, Westover MB (2019) Automated tracking of level of consciousness and delirium in critical illness using deep learning. NPJ digital medicine 2:89. doi:10.1038/s41746-019-0167-0

6. Allahyari H, Deisenhammer E, Weiser G (1976) EEG examination during delirium tremens. Psychiatria clinica 9 (1):21-31. doi:10.1159/000283662

7. Fleischmann R, Tränkner S, Bathe-Peters R, Rönnefarth M, Schmidt S, Schreiber SJ, Brandt SA (2019) Diagnostic Performance and Utility of Quantitative EEG Analyses in Delirium: Confirmatory Results From a Large Retrospective Case-Control Study. Clinical EEG and neuroscience 50 (2):111-120. doi:10.1177/1550059418767584

8. Kimchi EY, Neelagiri A, Whitt W, Sagi AR, Ryan SL, Gadbois G, Groothuysen D, Westover MB (2019) Clinical EEG slowing correlates with delirium severity and predicts poor clinical outcomes. Neurology 93 (13):e1260-e1271. doi:10.1212/wnl.0000000000008164

9. Numan T, van den Boogaard M, Kamper AM, Rood PJT, Peelen LM, Slooter AJC (2019) Delirium detection using relative delta power based on 1-minute single-channel EEG: a multicentre study. Br J Anaesth 122 (1):60-68. doi:10.1016/j.bja.2018.08.021

10. Matsushima E, Nakajima K, Moriya H, Matsuura M, Motomiya T, Kojima T (1997) A psychophysiological study of the development of delirium in coronary care units. Biological psychiatry 41 (12):1211-1217. doi:10.1016/s0006-3223(96)00219-3

11. Vacas S, McInrue E, Gropper MA, Maze M, Zak R, Lim E, Leung JM (2016) The Feasibility and Utility of Continuous Sleep Monitoring in Critically Ill Patients Using a Portable Electroencephalography Monitor. Anesthesia and analgesia 123 (1):206-212. doi:10.1213/ane.0000000000001330

12. Trzepacz PT, Sclabassi RJ, Van Thiel DH (1989) Delirium: a subcortical phenomenon? The Journal of neuropsychiatry and clinical neurosciences 1 (3):283-290. doi:10.1176/jnp.1.3.283

13. Katz IR, Mossey J, Sussman N, Muenz L, Harner R, Curlik S, Sands LP (1991) Bedside clinical and electrophysiological assessment: assessment of change in vulnerable patients. International psychogeriatrics 3 (2):289-300. doi:10.1017/s104161029100073x

14. Fleischmann R, Traenkner S, Kraft A, Schmidt S, Schreiber SJ, Brandt SA (2019) Delirium is associated with frequency band specific dysconnectivity in intrinsic connectivity networks: preliminary evidence from a large retrospective pilot case-control study. Pilot and feasibility studies 5:2. doi:10.1186/s40814-018-0388-z

15. Thomas C, Hestermann U, Kopitz J, Plaschke K, Oster P, Driessen M, Mundt C, Weisbrod M (2008) Serum anticholinergic activity and cerebral cholinergic dysfunction: an EEG study in frail elderly with and without delirium. BMC neuroscience 9:86. doi:10.1186/1471-2202-9-86

16. Thomas C, Hestermann U, Walther S, Pfueller U, Hack M, Oster P, Mundt C, Weisbrod M (2008) Prolonged activation EEG differentiates dementia with and without delirium in frail elderly patients. Journal of neurology, neurosurgery, and psychiatry 79 (2):119-125. doi:10.1136/jnnp.2006.111732

17. Nielsen RM, Urdanibia-Centelles O, Vedel-Larsen E, Thomsen KJ, Møller K, Olsen KS, Lauritsen A, Eddelien HS, Lauritzen M, Benedek K (2020) Continuous EEG Monitoring in a Consecutive Patient Cohort with Sepsis and Delirium. Neurocritical care 32 (1):121-130. doi:10.1007/s12028-019-00703-w

18. Knauert MP, Gilmore EJ, Murphy TE, Yaggi HK, Van Ness PH, Han L, Hirsch LJ, Pisani MA (2018) Association between death and loss of stage N2 sleep features among critically Ill patients with delirium. Journal of critical care 48:124-129. doi:10.1016/j.jcrc.2018.08.028

19. Jacobson SA, Leuchter AF, Walter DO (1993) Conventional and quantitative EEG in the diagnosis of delirium among the elderly. Journal of neurology, neurosurgery, and psychiatry 56 (2):153-158. doi:10.1136/jnnp.56.2.153

20. Jacobson SA, Leuchter AF, Walter DO, Weiner H (1993) Serial quantitative EEG among elderly subjects with delirium. Biological psychiatry 34 (3):135-140. doi:10.1016/0006-3223(93)90382-n

21. Reischies FM, Neuhaus AH, Hansen ML, Mientus S, Mulert C, Gallinat J (2005) Electrophysiological and neuropsychological analysis of a delirious state: the role of the anterior cingulate gyrus. Psychiatry research 138 (2):171-181. doi:10.1016/j.pscychresns.2004.06.008

22. Numan T, Slooter AJC, van der Kooi AW, Hoekman AML, Suyker WJL, Stam CJ, van Dellen E (2017) Functional connectivity and network analysis during hypoactive delirium and recovery from anesthesia. Clinical neurophysiology : official journal of the International Federation of Clinical Neurophysiology 128 (6):914-924. doi:10.1016/j.clinph.2017.02.022

23. Plaschke K, Hill H, Engelhardt R, Thomas C, von Haken R, Scholz M, Kopitz J, Bardenheuer HJ, Weisbrod M, Weigand MA (2007) EEG changes and serum anticholinergic activity measured in patients with delirium in the intensive care unit. Anaesthesia 62 (12):1217-1223. doi:10.1111/j.1365-2044.2007.05255.x

24. van Dellen E, van der Kooi AW, Numan T, Koek HL, Klijn FA, Buijsrogge MP, Stam CJ, Slooter AJ (2014) Decreased functional connectivity and disturbed directionality of information flow in the electroencephalography of intensive care unit patients with delirium after cardiac surgery. Anesthesiology 121 (2):328-335. doi:10.1097/aln.0000000000000329

25. Koponen H, Partanen J, Pääkkönen A, Mattila E, Riekkinen PJ (1989) EEG spectral analysis in delirium. Journal of neurology, neurosurgery, and psychiatry 52 (8):980-985. doi:10.1136/jnnp.52.8.980

26. Keijzer HM, Klop M, van Putten M, Hofmeijer J (2020) Delirium after cardiac arrest: Phenotype, prediction, and outcome. Resuscitation 151:43-49. doi:10.1016/j.resuscitation.2020.03.020

27. van der Kooi AW, Zaal IJ, Klijn FA, Koek HL, Meijer RC, Leijten FS, Slooter AJ (2015) Delirium detection using EEG: what and how to measure. Chest 147 (1):94-101. doi:10.1378/chest.13-3050

28. Hunter A, Crouch B, Webster N, Platt B (2020) Delirium screening in the intensive care unit using emerging QEEG techniques: A pilot study. AIMS neuroscience 7 (1):1-16. doi:10.3934/Neuroscience.2020001

29. Watts DJ, Strogatz SH (1998) Collective dynamics of 'small-world' networks. Nature 393 (6684):440-442. doi:10.1038/30918

30. Kaminski M, Blinowska KJ (2018) Is Graph Theoretical Analysis a Useful Tool for Quantification of Connectivity Obtained by Means of EEG/MEG Techniques? Frontiers in neural circuits 12:76. doi:10.3389/fncir.2018.00076
